# Low use of long-lasting insecticidal nets for malaria prevention in south-central Ethiopia: A community-based cohort study

**DOI:** 10.1371/journal.pone.0210578

**Published:** 2019-01-10

**Authors:** Tarekegn Solomon, Eskindir Loha, Wakgari Deressa, Taye Gari, Hans J. Overgaard, Bernt Lindtjørn

**Affiliations:** 1 School of Public Health, College of Medicine and Health Sciences, Hawassa University, Hawassa, Ethiopia; 2 Centre for International Health, University of Bergen, Bergen, Norway; 3 Department of Preventive Medicine, School of Public Health, College of Health Sciences, Addis Ababa University, Addis Ababa, Ethiopia; 4 Faculty of Science and Technology, Norwegian University of Life Sciences, Akershus, Norway; Ministry of Health and Sports, MYANMAR

## Abstract

**Introduction:**

A decline in malaria morbidity and mortality has been documented in Ethiopia since 2005 following a scale-up of the distribution of long-lasting insecticidal nets (LLINs). However, universal access to LLINs ownership and use has not yet been achieved. This study aimed to determine ownership and use of LLINs over time in south-central Ethiopia.

**Methods:**

A cohort of 17,142 individuals residing in 3,006 households was followed-up from October 2014 to January 2017 (121 weeks). New PermaNet2.0 LLINs were given to households in October 2014. Once per week, the LLIN use status was documented for each individual. A survey was conducted after 110 weeks of LLIN distribution to determine LLIN ownership. A multilevel negative binomial regression model was fitted to identify significant predictors of LLIN use.

**Results:**

At baseline, the LLIN ownership was 100%. After 110 weeks only 233 (8%) of the households owned at least one LLIN. The median proportion of LLIN use per individuals during the study period was only 14%. During the first year (week 1–52) the average LLIN use per individuals was 36% and during the second year (week 53–104) it was 4.6%. More frequent LLIN use was reported among age group [5–14 years (adjusted IRR = 1.13, 95% CI 1.04–1.22), 15–24 years (adjusted IRR = 1.33, 95% CI 1.23–1.45), ≥25 years (adjusted IRR = 1.99, 95% CI 1.83–2.17)] compared to <5 years, and household head educational status [read and write (adjusted IRR = 1.17, 95% CI 1.09–1.26), primary (adjusted IRR = 1.20, 95% CI 1.12–1.27), secondary or above (adjusted IRR = 1.20, 95% CI (1.11–1.30)] compared to illiterate. Having a family size of over five persons (adjusted IRR = 0.78, 95% CI 0.73–0.84) was associated with less frequent use of LLINs compared to a family size of ≤5 persons.

**Conclusions:**

The study showed a low LLIN ownership after 110 weeks and a low LLIN use during 121 weeks of follow-up, despite 100% LLIN coverage at baseline. The study highlights the need to design strategies to increase LLIN ownership and use for setting similar to those studied here.

## Introduction

A decline in malaria morbidity and mortality has been documented in sub-Saharan Africa since 2000, where an estimated 90% of global malaria cases and deaths have occurred [1, 2]. The use of the long-lasting insecticidal nets (LLINs) and indoor residual spraying (IRS) are considered the two main vector control interventions that played a role in the reduction of the malaria burden. Studies from sub-Saharan Africa showed that the use of LLINs alone has reduced malaria incidence rates by 50% and malaria mortality rates by 55% in children under the age of 5 years [3, 4]. Moreover, in the past 15 years increases in LLIN coverage and use have been documented in sub-Saharan Africa [2]. Although a decline in malaria burden and increases in LLIN access and use were documented, the use of LLINs by people at risk remained lower than expected [5]. For example, in 2016, only 43% of people had access to sufficient LLINs (one net for two people), and only 54% of people at risk for malaria used LLINs [5]

In Ethiopia, where 60% of the population are at risk of malaria infection, and 68% of the country’s area is favourable for malaria transmission, a decline in malaria morbidity and mortality has been observed since 2005 [6]. A scaling-up of anti-malaria interventions, such as LLINs, IRS, malaria diagnoses using a rapid diagnostic test (RDT) and prompt treatment using artemether-lumefantrine, are believed to be the primary reasons for these achievements [7, 8]. An estimated 64 million LLINs were distributed within the country through periodic mass campaigns between 2005 and 2015 [9], with an additional 29.6 million LLINs distributed in 2015 [10]. Moreover, Ethiopia had set a target to achieve 100% of LLIN coverage (at least one LLIN per sleeping space in malaria-endemic areas) and 80% of use (people at risk of malaria shall use LLINs properly and consistently) by the year 2015 [11]. However, the three national malaria indicator surveys (MIS) have shown that universal access to LLIN ownership and use has yet to be achieved. The households with at least one LLIN were 65% in 2007, 55% in 2011 and 64% in 2015 [12–14]. Furthermore, households with at least one LLIN for every two persons were as low as 37% in 2007, 24% in 2011 and 32% in 2015 [12–14]. The overall LLIN use rates were 32% in 2007 and 40% in 2015 MIS surveys [12, 14]. The commonly reported barriers to the use of LLINs in Ethiopia include worn out LLINs, a lack of space to hang the LLINs, living away from vector breeding sites, discomfort, a low-risk perception of malaria, saving nets for future use, and a lack of awareness and perception of low efficacy to prevent malaria [15–17].

To achieve the goals and targets for reducing the malaria burden, consistent use of LLINs by people at risk of malaria must be maintained. Therefore, understanding the level of LLINs ownership and use over time is helpful to evaluate existing strategies and subsequent LLINs distribution campaigns. Previous studies in Ethiopia evaluate the LLIN ownership and use using cross-sectional study designs [12, 14, 16, 18]. Because of the nature of the designs, they fail to show trends of LLIN use over time after mass LLIN distributions campaigns. To fill this knowledge gap, a prospective cohort study design was used to evaluate LLIN use of each study participant for more than two years (121 weeks). The weekly follow-up started in October 2014 immediately after distribution of LLINs free of charge according to the National Malaria Guidelines [19]. Therefore, the aim of this study was to determine the LLIN ownership and use over time, and to identify factors associated with LLIN use in south-central Ethiopia.

## Methods

### Ethical clearance

Ethical clearance was obtained from Ethiopian Ministry of Science and Technology (Ref: 3.10/446/06), Institutional Review Board (IRB) of the College of Health Sciences of Addis Ababa University, and the Regional Committee for Medical and Health Research Ethics, Western Norway (Ref: 2013/986/REK vest). Permission letters were obtained from the Oromia Regional Health Bureau, the East Shewa Zonal Health Department and the Adami Tullu District Health Office. Before the start of the study, the community elders, *Kebele* and village leaders were sensitized about the study objectives, implementation processs and expected outcomes of the study. Verbal informed consent was obtained from the head of household or members of the household older than 18 years in the absence of the head of household. We opted to take verbal informed consent because we had a challenge to get written consent as the majority of the study participants could not read and write [20]. We used a standard information sheet to explain the purpose of the study. The participants were informed that participation was voluntary and they had the right to withdraw from the study at any time. The information was read to study participants using the information sheet in their own language (*Afan Oromo*), and their consent was recorded using check mark.

### Study setting

**Fig 1:**
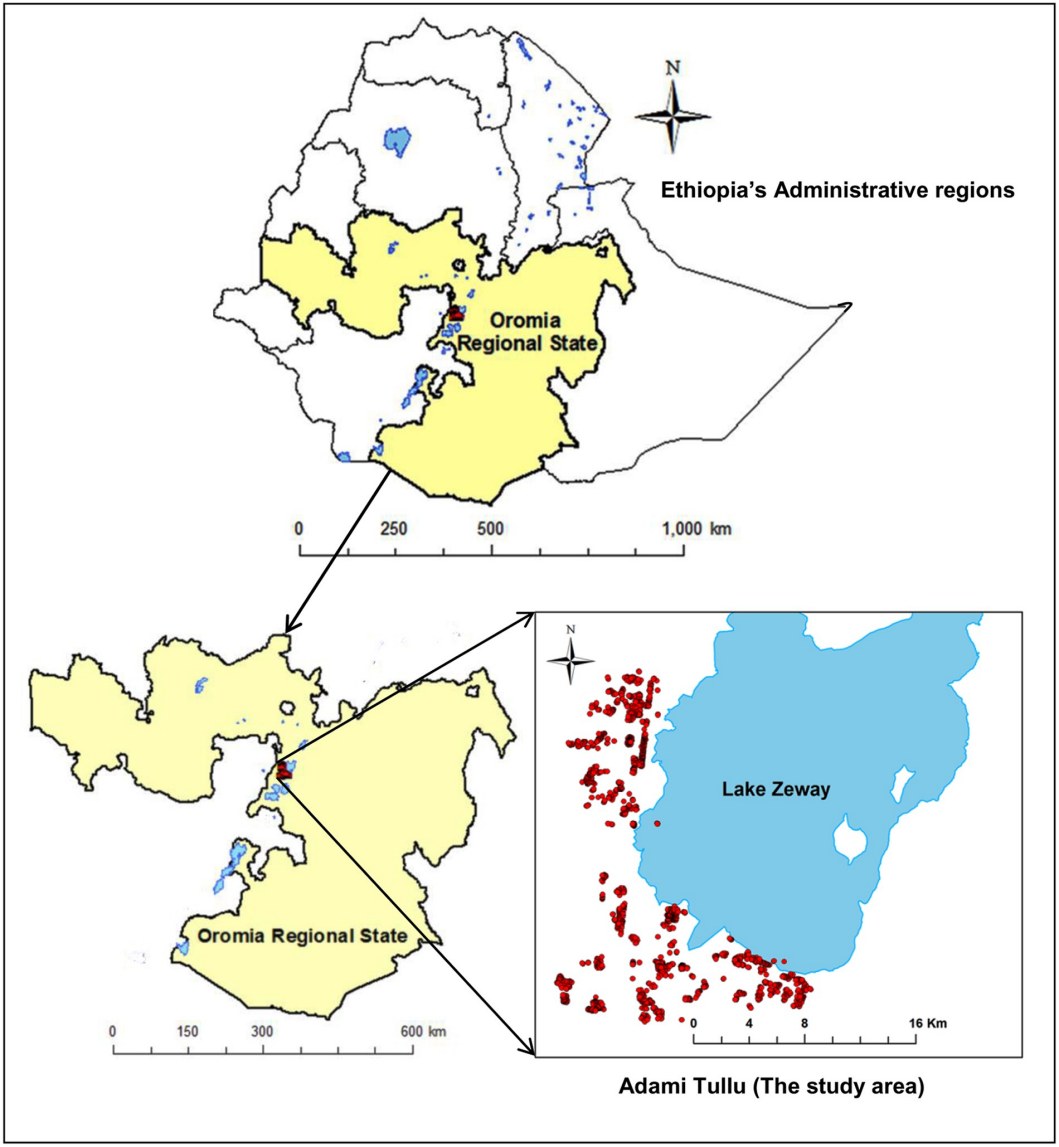
Map of the location of the study households in the Adami Tullu District in south-central Ethiopia.

This study was conducted in the Adami Tullu District of Oromia Regional State in south-central Ethiopia (Fig 1) from October 2014 to January 2017. The district is situated in the East African Great Rift Valley, approximately 160 km south of Addis Ababa. Based on the 2007 national census, approximately 190,000 people lived in the district in 2017 [21]. The majority of the population live in rural areas, and are engaged in subsistence farming and livestock rearing. The district is characterized by a semi-arid climatic condition, with a total annual precipitation of 700 mm, an average minimum temperature of 14.5 °C and a maximum temperature of 27.7 °C [20].

Malaria is among the leading causes of morbidity and mortality in the district. Malaria transmission is seasonal, and peaks during the months from September to December following the monsoon rains in July and August [19]. The shores and irrigated areas around Lake Zeway serve as mosquito breeding sites [22, 23]. The primary malaria vector is *Anopheles arabiensis*. *Plasmodium falciparum* and *Plasmodium vivax* are the two main malaria parasites causing malaria infection [24, 25]. During the study period, a 63% decline in malaria incidence was reported [26]. In this period, the district experienced a severe drought and food shortage following the El Nino of 2015 [27].

### Study design and participants

This study was part of a cluster-randomized controlled trial that aimed to determine whether the combined use of LLINs and IRS with propoxur provides additional protection against malaria (*Plasmodium falciparum* and/or *Plasmodium vivax*) among all age groups compared to LLINs or IRS alone (the Maltrials project). The trial is described in more detail in the published protocol [20]. Briefly, the unit of randomization was villages (clusters) that contained approximately 35 households and 196 people in each cluster. A total of 176 clusters (44 clusters per arm) from 13 kebeles (the lowest government administrative unit) were included in the trial. Clusters were identified based on the rate of malaria transmission and located within 5 km from Lake Zeway. The trial distributed in all 7,740 PermaNet2.0 LLINs (Vestergaard Frandsen Group) free of charge to 3,006 households (4,157 LLINs in the combined LLIN+IRS arm and 3,583 LLINs in the LLIN arm), with an average of 2.57 LLINs per household. The LLINs had a light blue colour and rectangular shape, with a width of 160 cm, a length of 180 cm and a height of 150 cm. Although we planned for 104 weeks (two complete years) of follow-up, the period was extended to 121 weeks to add one additional malaria transmission season; thus including three complete transmission seasons. The reason for adding the third season was to increase the number of cases, because malaria was lower than expected, potentially due to a draught caused by El Nino of 2015 [27].

The number of LLINs distributed to each household was recorded at baseline. Following the National Malaria Guidelines [19] one LLIN was given to a family of 1–2 persons; two LLINs to a family of 3–5 persons; three LLINs to a family of 6–7 persons and four LLINs to a family with ≥ 8 persons. Two weeks after LLINs distribution, a “hang-up” campaign was carried out by fieldworkers, which consisted of education on LLIN handling and proper use.

**Fig 2:**
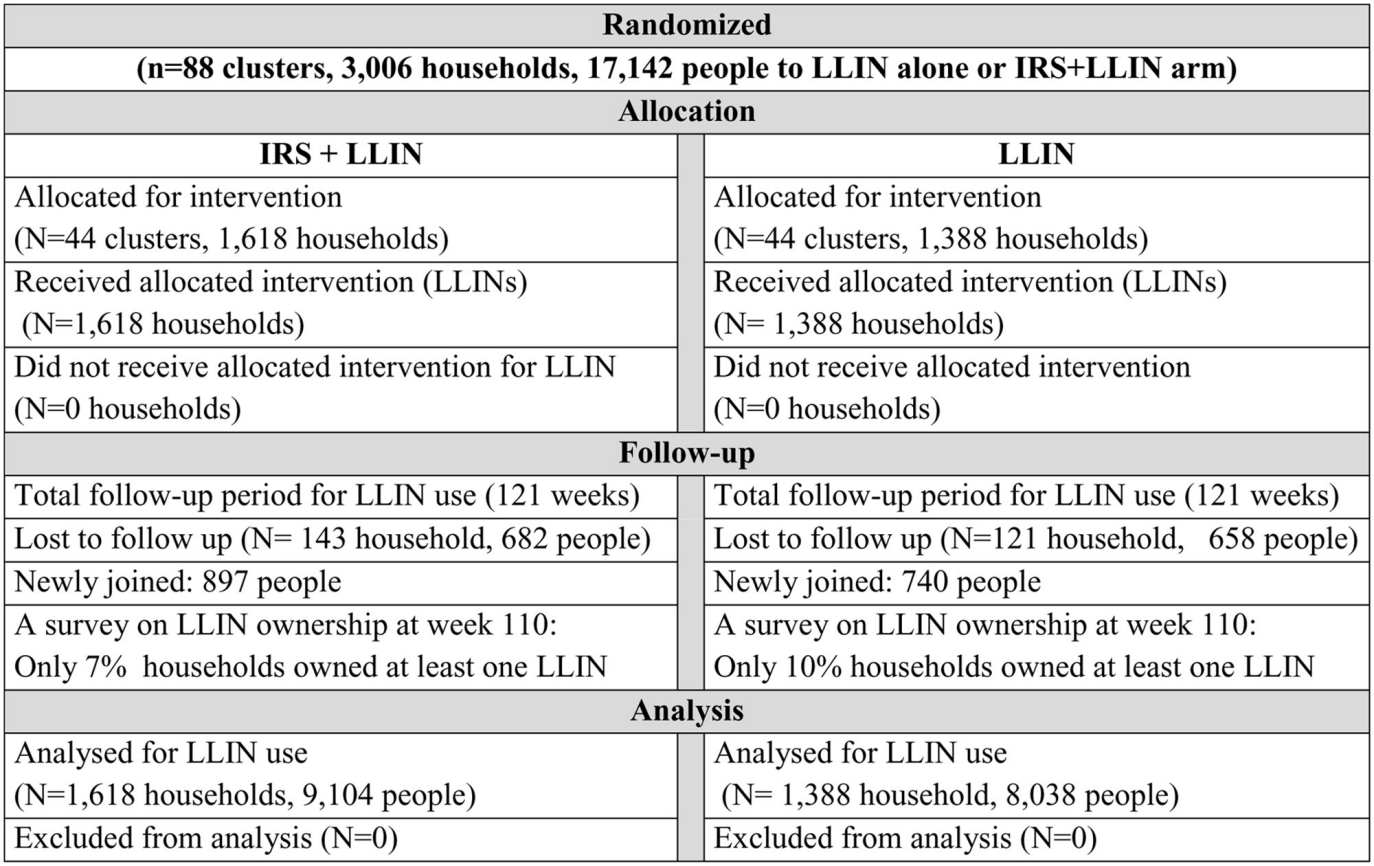
Flow chart of the study for weekly long-lasting insecticidal nets use in Adami Tullu District south-central Ethiopia, October 2014 to January 2017.

A cohort study was conducted among 17,142 people in the 3,006 households of the LLIN+IRS and LLIN arms to quantify the LLIN use. All study participants were followed on a weekly basis for 121 weeks, from October 2014 to January 2017. All study participants were followed until the end of the study or until they were lost to follow-up. Newcomers (individuals who joined a cohort as new household members) and newborns during the study period were included in the study (Fig 2). A cross-sectional survey was carried out at the 110^th^ week post-distribution to assess LLIN ownership among all households that received LLINs at baseline and to validate the results of LLIN use. A parallel follow-up study was conducted from October 2014 to November 2016 in the sampled households aiming to assess attrition, physical integrity, functional survival and bio-efficacy of the LLINs. The results of the parallel study are reported elsewhere [28]

### Data collection

A baseline census was conducted in August 2014 using pre-tested interviewer-administered questionnaires containing socio-demographic and economic variables (see S1 File). The questionnaires were prepared in English, and then translated into *Afan Oromo* (local language). Two more such censuses were carried out to update the follow-up population at weeks 51 and 103.

We carried out weekly home visits to record the LLIN use of the study participants (see S2 File). Each week, the heads of households or family members aged ≥18 years were asked whether any household members used an LLIN the night before the day of the interview. The names and codes of the individuals who used the LLIN were recorded. If the visited houses were closed, or if heads of households or family members aged ≥18 years were absent, the data collectors visited the house at least three more times within the same week. If one or more or all of the household members had left the study area during the study period, the individuals were considered lost to follow-up. In subsequent follow-ups, the households were visited on the same day of the week to maintain a seven-day gap between visits. The visits were carried out early in the morning to observe whether the LLINs were hung in the sleeping space. Moreover, during weekly follow-up for the trial study [20], data collectors identified and referred people with a history of fever over the past 48 hours to health post for malaria diagnosis. The families were advised to visit the health post if any family member developed fever between the dates of home visits. Individuals who were found to be positive for malaria parasites were treated according to national guidelines [19].

For the LLIN ownership survey, respondents were asked if they had useable LLINs in their household. The presence of usable LLINs was verified in the visited household by observation. If the LLINs were lost, the reasons for the loss were asked.

Twenty-four data collectors having a college diploma were recruited from the respective kebeles. Three supervisors were recruited to monitor the overall data collection process, and to control data quality. The data collectors and supervisors were trained for five days on the use of questionnaires, interviewing techniques, household visits and supervision. The data collection questionnaire for weekly LLIN use was adopted from a longitudinal study from southern Ethiopia [15] and from a pilot study in the study area [25]. The questionnaires were cross-checked for their reliability with source households by the supervisors.

### Statistical analysis

A total of 17,142 study participants in 3,006 households were included in the analysis. For non-normally distributed continuous variables, medians and interquartile range (IQR) were calculated. The ownership of LLINs after two years was calculated by taking the number of households with at least one LLIN as the numerator and the total number of households enrolled in the study at baseline as the denominator, excluding the number of households lost to follow-up from the denominator. To calculate the proportion of individuals using LLIN per week, we used the total number of individuals in all households who used an LLIN the night before the day of the interview as the numerator and the total population in all households of that week as the denominator as shown in the following formula.

PoportionofindividualsusingLLINperweek=TotalnumberofindividualssleptunderLLINsineachweekTotalpopulationoftheweek×100%

**Fig 3:**
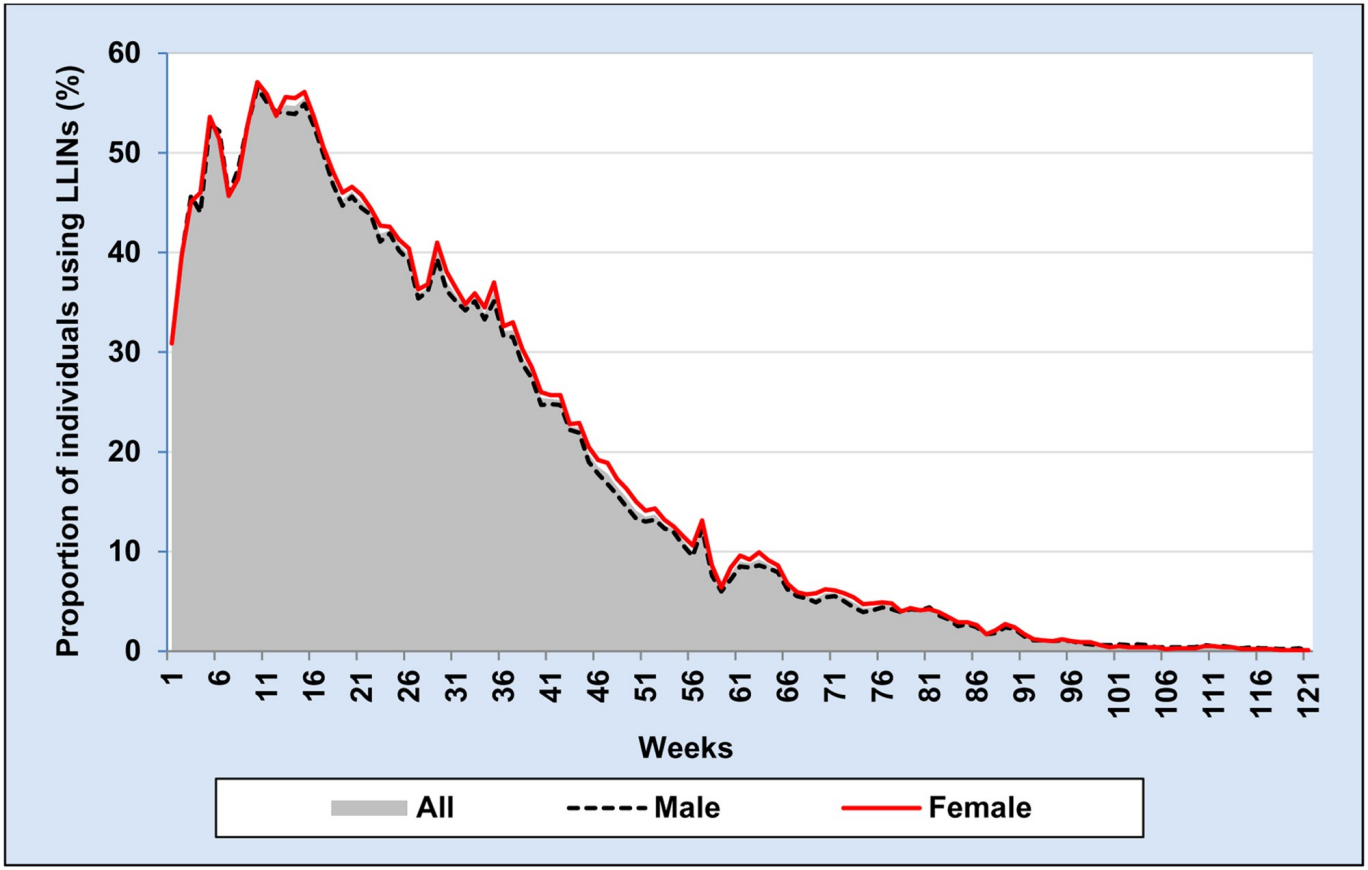
Weekly proportion of individuals using long-lasting insecticidal net by gender during 121 weeks from October 2014 to January 2017.

**Fig 4:**
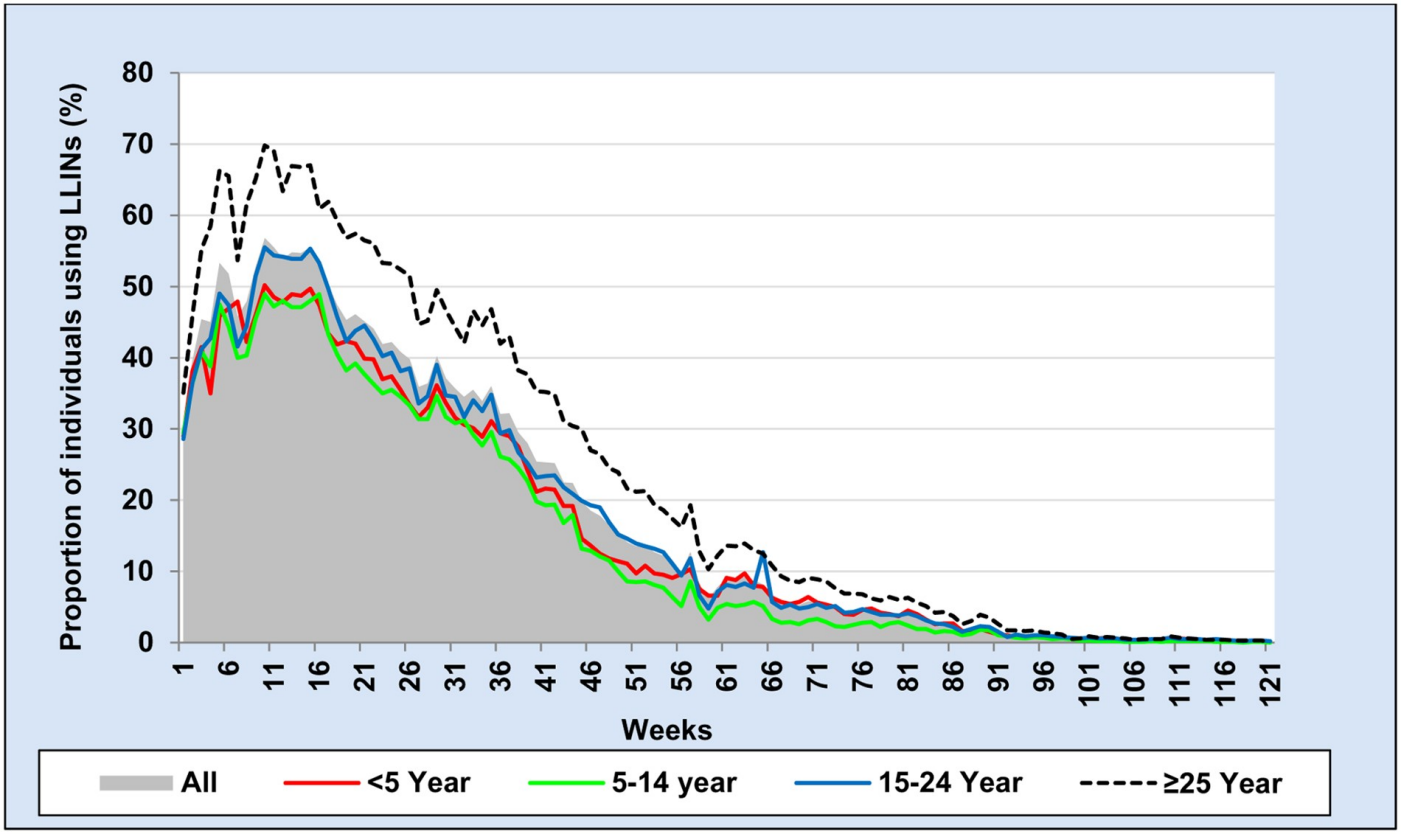
Weekly proportion of individuals using long-lasting insecticidal net by age group during 121 weeks from October 2014 to January 2017.

**Fig 5:**
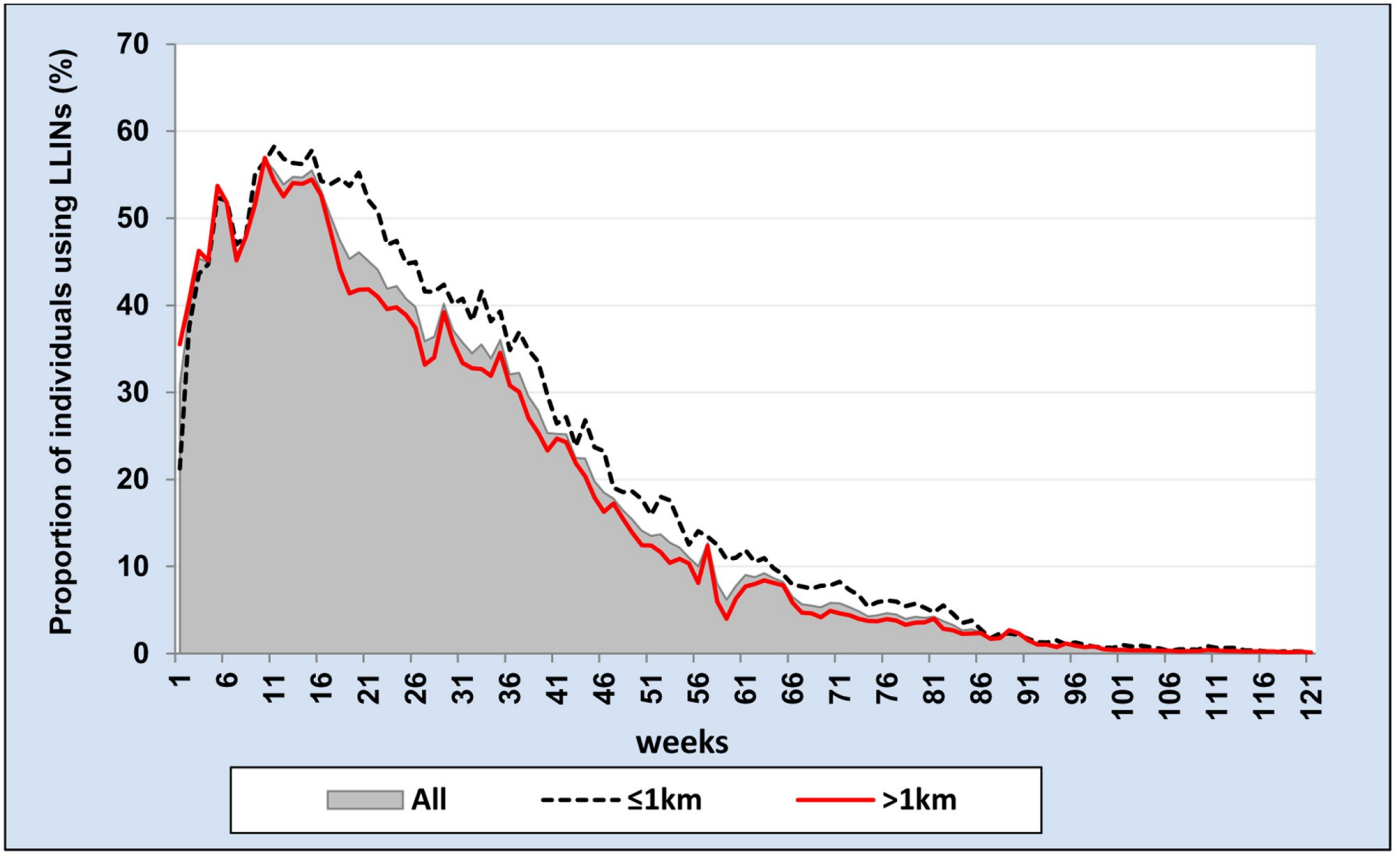
Weekly proportion of individuals using long-lasting insecticidal net by distance from potential vector breeding site during 121 weeks from October 2014 to January 2017.

The proportion of individuals using LLIN per week was calculated for each week of 121 weeks and stratified by gender, age groups and distance of household from potential vector breeding sites (see Figs 3, 4 and 5). To calculate the proportion of LLIN use per each individual per the whole study period, we used the total number of weeks in which LLIN use was reported by each individual during the study period as the numerator and the total number of weeks an individual stayed in the study area as the denominator as shown in the following formula.

ProportionofLLINuseperindividual=TotalnumberofweeksanindividualreportedLLINuseduringthestudyperiodTotalnumberofweeksanindividualstayedinthestudyarea×100%

The median and interquartile range (IQR) proportion of LLIN use per individuals were calculated and reported to all 17,142 study participants.

**Fig 6:**
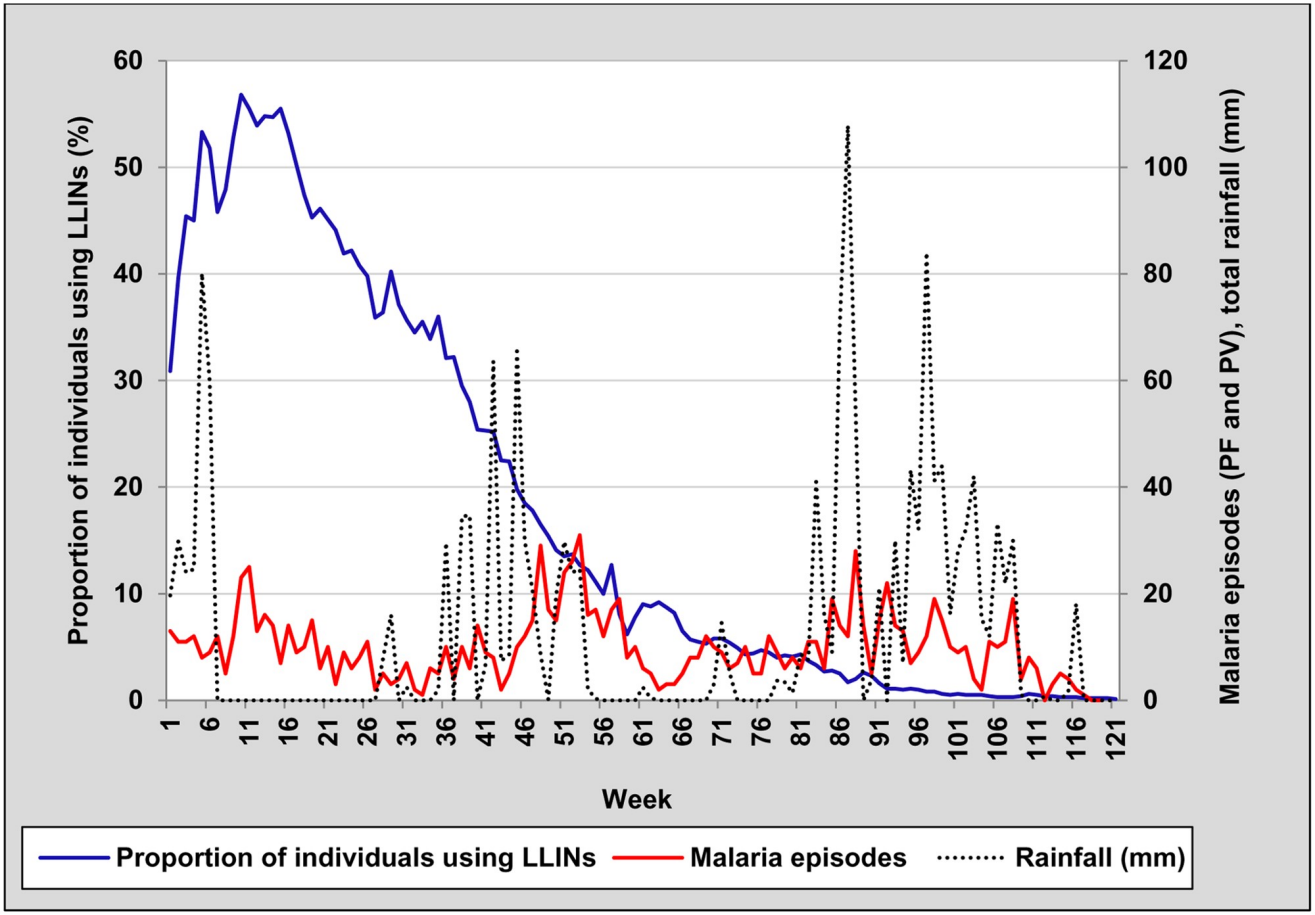
Sequence chart of weekly proportion of individuals using long-lasting insecticidal nets (LLINs) (lagged by 2 weeks), malaria episodes [plasmodium Falciparum (PF) and P. Vivax (PV)], and total rainfall (lagged by 6 weeks), south-central Ethiopia, October 2014-January 2017.

We used weekly malaria episodes collected as part of the main trial [20], rainfall and proportion of individuals using LLINs per week data to construct a sequence chart, similar to that of Loha et al. [29], to show seasonal patterns of proportion of individuals using LLINs per week (lagged by 2 weeks—considering the incubation period of malaria infection) compared with malaria episodes and rainfall (lagged by 6 weeks—as in the model published from relatively similar setup) (see Fig 6).

A principal component analysis (PCA) was used to calculate the household wealth index [30, 31]. Fourteen household assets were included in the calculation: presence of electricity, ownership of a television, radio, mobile telephone, chair, table, bed, bicycle, land, a separate kitchen from the house, livestock and cart, as well as type of roof and wall. The first principal component was taken to construct the wealth index. Next, the index values were categorized into three relative measures of socioeconomic classes, poor, middle-class and rich. The details of wealth index calculations are reported elsewhere [32]. Distance from potential vector breeding sites to a household (in km) was calculated using proximity analysis in ESRI ArcMap 10.3 (CA, USA). The buffer option under proximity analysis was used to categorize the distance of households into ≤1 km and >1 km from the border line around Lake Zeway or the Bulbula River.

To investigate the predictors of LLIN use, we ran both a Poisson regression and a negative binomial regression models separately. When the Poisson regression model was fitted to the count data of total number of weeks in which LLIN use was reported, the ratio of the deviance over the degree of freedom was 13.8. This value became 1.2 when a negative binomial regression model was fitted. Since the latter model handled the problem of overdispersion, we used negative binomial regression model as the final model. Furthermore, to account for clustering effect of LLIN use within individual, household and village level, we used a multilevel negative binomial regression model. We assumed that the predictors of LLIN use were clustered at three levels. Individuals (first level) were nested within households with the assumption that individuals have similar LLIN use within a household, but different LLIN use between households. Similarly, households (second level) were nested within villages (third level) with the assumption that LLIN use was similar among households within a village, but different between villages. Based on these assumptions, the presence of clustering was checked before fitting a multilevel negative binomial regression model. The following steps were used to check clustering: first, a null single level (standard) negative binomial regression model was fitted to the data, and then a null multilevel negative binomial regression with the random household and village effect was fitted. The estimated variance for village level random effect was 0.58 (SE = 0.24); for household level 0.24 (SE = 0.06). The calculated likelihood ratio test statistics showed strong evidence of village and household effect on the LLIN use (Chi-square = 5627.38, P<0.001). Therefore, to account for the clustering effect, we used a multilevel negative binomial regression model to estimate unadjusted incidence rate ratio (IRR) and adjusted IRR of LLIN use with 95% confidence interval (CI). To construct the model, the following parameters were specified: total number of weeks in which study participants reported LLIN use as dependent variable; total number of weeks study participants stayed (followed-up) in the study area as exposure variable. The covariance structure of the random effects was unstructured and standard error type was robust. Age, gender, educational and occupational status of the head of the household, household wealth status, household size, number of sleeping spaces in the household, study arm, and distance from a lake or river were considered as independent variables for LLIN use. Independent variables having a P-value <0.25 in bivariate analysis were included in the multivariate to identify significant predictors of LLIN use, adjusting for other variables. The level of statistical significance was set as a P-value <0.05. Data were entered into SPSS version 20.0 (Armonk, NY: IBM Corp. USA) and, analyzed using both SPSS and STATA version 15 (StataCorp, Texas, USA).

## Results

### Characteristics of the study participants

The median follow-up time was 121 weeks, and the median population size was 6.0 (IQR: 4–7) with a range of 1 to 18 people per household. Approximately 1,650 (54.9%) of the head of households were illiterate. Approximately one-third, 1,006 (33.5%) of the households were located within 1 km from potential vector breeding sites close to Lake Zeway or the Bulbula River (Table 1).

**Table 1:** Characteristics of the study participants and their households, Ethiopia.

Variable	n (%)
**Gender (n = 17,142)**	
Male	8,618 (50.3)
Female	8,524 (49.7)
**Age in years (n = 17,132)**	
<5	3,196 (18.7)
5–14	5,557 (32.4)
15–24	3,396 (19.8)
≥25	4,983 (29.1)
**Educational status of head of household**^a^	
Illiterate	1,650 (54.9)
Can read and write	274 (9.1)
Primary	759 (25.2)
Secondary and above	323 (10.7)
**Occupational status of head of household**^a^	
Farmer	2,278 (75.8)
Others	728 (24.2)
**Household size**^a^	
≤5 persons	1,449 (48.2)
>5 persons	1,557 (51.8)
**Wealth status**^a^	
Poor	1,153 (38.4)
Medium	978 (32.5)
Rich	875 (29.1)
**Intervention arm**^a^	
LLIN+IRS	1,618 (53.8)
LLIN alone	1,388 (46.2)
**Distance from lake or river**^a^	
≤1km	1,006 (33.5)
>1km	2,000 (66.5)

^a^ calculated for household characteristics (n = 3006 households)

### Long-lasting insecticidal net ownership

In October 2014, a total of 7,740 PermaNet2.0 LLINs were distributed to 3,006 households free of charge by the Maltrials project (with an average of 2.57 LLINs per household). After two years in November 2016 (week 110), 2,788 (93%) households were surveyed to determine whether the distributed LLINs were available or not. In that survey, 218 (7%) households were not evaluated, as the houses were closed or the household members had moved to other locations. Only 233 (8%) of the interviewed households had at least one LLIN. The remaining 2,555 (92%) households had lost their LLINs (Table 2).

**Table 2:** Number and percentage of households with long-lasting insecticidal nets at baseline and after 110 weeks, Ethiopia November 2016.

Number of LLINs available per household	Week 1 (baseline) (n = 3,006 households)		Week 110 (n = 2,788 households)*		LLINs ownership at week 110 compared to the baseline
	Number	Percent	Number	Percent	P-value^‡^
0	0	0.0	2,555	91.6	
1	423	14.1	182	6.5	<0.001
2	1,275	42.4	45	1.6	<0.001
3	799	26.6	6	0.2	<0.001
4	509	16.9	0	0.0	<0.001
1–4	3,006	100.0	233	8.4	<0.001

*218 households were not evaluated at survey in week 110 due to being closed or moved to other location.

LLINs = long-lasting insecticidal nets

^‡^Z-test was used to compare proportions of LLIN ownership

### Reasons for long-lasting insecticidal net loss

The most common reason for LLIN loss (76%; n = 4713) was that the LLINs were thrown away due to damage. The second most common (12%; n = 750) reason for LLIN loss was the misuse of LLINs for other purposes, such as grain transportation from the field, grain storage at home and toilet cover. Some (9%; n = 554) reported that they gave the LLINs to their children when they sent them to school or gave them to relatives living in other places (Table 3).

**Table 3:** Reported causes for long-lasting insecticidal net loss after two years of post-distribution, Ethiopia November 2016.

Reason for LLIN loss	Number	Percentage
Thrown away	4,713	75.7
Used for something else	750	12.0
Given away	554	9.0
Stolen	84	1.3
Other*	73	1.2
Don’t know	52	0.8
Total	6,226	100.0

*reported as not receiving the LLIN at all or sold

LLIN = long-lasting insecticidal net

### Trend of long-lasting insecticidal net use

During 121 weeks of follow-up, the median proportion of LLIN use per individual was 14.0% (IQR: 4.1–29.8%) (Table 4). The mean proportion of LLIN use per individual during the first year (week 1–52) was 36% and in the second year (week 53 to 104) it was just 4.6%. The proportion of individuals using LLIN per week among females was slightly higher than males (Table 4 and Fig 3). In general, weekly proportion of individuals using LLIN was higher among age group 15–24 years and older than 25 years compared to other age groups (Table 4 and Fig 4). Individuals who were living in the wealthier households reported higher proportion of LLIN use per individual compared with the poor (Table 4). Similarly, people who were living within 1 km from potential mosquito breeding sites reported a higher weekly proportion of LLIN use compared to those who were living further away than 1 km (Table 4 and Fig 5). However, gender, intervention arm, wealth index and distance from potential vector breeding site were not significantly associated with the total number of LLIN use report during the study period after multilevel analysis, adding random effect variables at household and village level (Table 5).

**Table 4:** The median (IQR) proportion of weeks individuals used an LLIN during 121 weeks from October 2014 to January 2017.

Variables	n	Median (IQR)	t- test*	P-value
**Gender**				
Male	8,618	13.2 (4.1–28.9)	Ref	
Female	8,524	14.0 (4.1–30.6)	1.97	0.048
**Age in years**				
<5	3,196	9.1 (0.8–24.0)	Ref	
5–14	5,557	9.9 (3.3–24.8)	1.84	0.066
15–24	3,396	13.2 (4.1–28.1)	8.29	<0.001
≥25	4,983	23.1 (10.7–35.5)	30.67	<0.001
**Intervention arm**				
LLIN+IRS	9,104	13.2 (4.1–29.8)	Ref	
LLIN alone	8,038	14.0 (4.1–29.8)	1.97	0.049
**Wealth index**				
Poor	6,058	12.4 (2.5–28.9)	Ref	
Medium	5,671	14.9 (5.8–30.6)	3.28	0.001
Rich	5,413	14.8 (5.8–30.6)	4.86	<0.001
**Distance from vector breeding**				
≤1 km	5,602	17.4 (3.3–30.6)	-11.93	<0.001
>1 km	11,540	12.4 (4.1–28.5)	Ref	
**All**	17,142	14.0 (4.1–29.8)^¥^		

* Test statistics was calculated using median regression model.

IQR = interquartile range, IRS = indoor residual spray, LLIN = long-lasting insecticidal net

^¥^ Overall mean (standard deviation) LLIN use = 17.8 (16.0), mean proportion of LLIN use per individuals during the first year (week 1–52) = 36%, and during the second year (week 53 to 104) = 4.6%.

**Table 5:** Multilevel negative binomial regression for predictors of long-lasting insecticidal net use, during 121 weeks from October 2014 to January 2017.

Variables	n (%)	Unadjusted IRR (95% CI)	P-value	Adjusted IRR (95% CI)	P-value
**Gender**					
Male	8,618 (50.3)	1			
Female	8,524 (49.7)	1.02 (0.99–1.04)	0.112	1.01 (0.98–1.03)	0.580
**Age (years) group**					
<5	3,196 (18.7)	1		1	
5–14	5,557 (32.4)	1.11 (1.02–1.20)	0.012	1.13 (1.04–1.22)	0.003
15–24	3,396 (19.8)	1.35 (1.24–1.46)	<0.001	1.33 (1.23–1.45)	<0.001
≥25	4,983 (29.1)	1.99 (1.83–2.16)	<0.001	1.99 (1.83–2.17)	<0.001
**Educational status of head of household**					
Illiterate	9,479 (55.3)	1		1	
Read and write	1,774 (10.3)	1.09 (1.02–1.17)	0.017	1.17 (1.09–1.26)	<0.001
Primary	4,281 (25)	1.15 (1.08–1.22)	<0.001	1.20 (1.12–1.27)	<0.001
Secondary and above	1,608 (9.4)	1.21 (1.11–1.32)	<0.001	1.20 (1.11–1.30)	<0.001
**Occupational status of head of household**					
Other	3,629 (21.2)	1			
Farmer	13,513 (78.8)	0.96 (0.91–1.01)	0.133	0.99 (0.93–1.05)	0.679
**Household size**					
≤5	5,212 (30.4)	1		1	
>5	11,930 (69.6)	0.72 (0.68–0.77)	<0.001	0.78 (0.73–0.84)	<0.001
**Number of sleeping spaces in household**					
1	3,843 (22.4)	1		1	
2	9,716 (56.7)	0.84 (0.78–0.89)	<0.001	0.94 (0.88–1.00)	0.054
≥3	3,583 (20.9)	0.81 (0.74–089)	<0.001	0.97 (0.89–1.05)	0.390
**Household wealth index**					
Poor	6,056 (35.3)	1		NA	
Medium	5,672 (33.1)	1.01 (0.93–1.10)	0.818		
Rich	5,414 (31.6)	1.01 (0.93–1.09)	0.895		
**Intervention arm**					
IRS+LLIN	9,104 (53.1)	1		NA	
LLIN alone	8,038 (46.9)	089 (0.65–1.22)	0.461		
**Distance from lake or river**					
≤1km	5,602 (32.7)	1		NA	
>1km	11,540 (67.3)	0.98 (0.72–1.35)	0.906		

IRR = incidence rate ratio, IRS = indoor residual spray, LLIN = long-lasting insecticidal net, NA = not applicable (P > 0.25 in bivariate analysis)

Fig 6 shows the pattern of LLIN use, malaria episodes and total rainfall in the follow-up period. Our study covered three main malaria transmission seasons which include: October—December 2014, September—December 2015 and September—December 2016. The proportion of individuals using LLIN per week was consistently declining in spite of seasonal variation of malaria and rainfall.

### Predictors of LLINs Use

Table 5 shows the association between the total number of weeks in which LLIN use reported and some explanatory variables. LLIN use was significantly higher in the age group from 5–14 years (adjusted IRR = 1.13, 95% CI 1.04–1.22), 15–24 years (adjusted IRR = 1.33, 95% CI 1.23–1.45) and ≥25 years (adjusted IRR = 1.99, 95% CI 1.83–2.17) compared with the age group <5 years. Similarly, LLIN use was higher among people whose heads of households could read and write (adjusted IRR = 1.17, 95% CI 1.09–1.26), had attended primary education (adjusted IRR = 1.20, 95% CI 1.12–1.27) and secondary or higher education (adjusted IRR = 1.20, 95% CI 1.11–1.30), compared with households where the household head was illiterate. On the other hand, people living in households with family size of more than five people (adjusted IRR = 0.78, 95% CI 0.73–0.84) were less likely to have used an LLIN compared to people living in households having a family size of five or less. In this study, gender, occupational status of head of household, number of sleeping spaces in household, household wealth status, distance from lake or river and interventional group did not show any significant difference.

## Discussion

A low LLIN use was observed in a semi-arid area of south-central Ethiopia. The median proportion of nights individuals used an LLIN during the 121 weeks follow-up period was only 14%. This low LLIN use could be explained by a high attrition rate of LLINs due to disposal, and misuse [33] of the LLINs for other purposes. Moreover, a low mosquito density and malaria incidence that occurred after a severe drought in 2015 in the study area could play an important role in the low use of LLINs [26, 34], since people may perceive lower risk of malaria infection and tend to use LLINs less likely in this condition.

This study was part of a large cluster-randomized control trial, in which we followed a large cohort of people for 121 weeks in a rural community of Ethiopia. Unlike cross-sectional studies on LLIN use, the weekly evaluation of LLIN use in this study gives the real LLIN use per week over the study period and during different seasons. Since the study population was randomly selected from a source population in a semi-arid area of south-central Ethiopia our findings can be generalized to many parts of Ethiopia that exhibit conditions similar to those in the study site.

In the current study, the ownership of LLINs was low despite universal LLIN coverage at baseline. Two years after distribution of LLINs, only 8% of the households owned at least one LLIN. LLIN ownership was lower compared with other studies in the area showing 27% ownership in 2013, 12% in 2014, and 31% in 2016 [25, 35, 36] and much lower than the 2015 national MIS, which reported 64% LLIN ownership [14]. Ownership was also lower than the study findings from Uganda [37, 38], Tanzania [39] and Madagascar [40]. The time interval between the LLIN distribution mass campaigns and data collection for each survey may be a reason for the observed difference in the level of LLIN ownership. Furthermore, a decline in mosquito population and malaria incidence that was observed in 2015 after a severe drought in the study area [26, 34], could be a potential reason for low LLINs ownership since people may perceive lower risk of malaria infection in such conditions.

In the current study, 92% of the households reported that they lost all the LLINs after two years of the distribution (Table 2). The most common reasons for LLIN loss were throwing away due to damage and using the LLINs for other purposes. A previous study in the same study area revealed that 21% of LLINs were lost after 6 months, 61% after 12 months and 96% after 24 months due to disposal because nets were damaged, torn or used for other purposes [28]. Studies from sub-Saharan African countries also reported similar reasons for LLINs loss [41, 42].

The observed mean LLIN use (36% over 52 weeks and 18% over 121 weeks of follow-up after 100% LLIN coverage) was lower than that of a previous longitudinal study from the Arba Minch area in southern Ethiopia (62% LLIN use over 49 weeks of follow-up after 98.4% LLIN coverage) [15]. The reasons for this difference may be related to the burden of malaria infection (14.7 malaria episodes per 1,000 persons per year in our study area versus 45.1 per 1,000 persons per year in the Arba Minch study area) [43]. Additionally, the observed first year mean LLIN use (36%) in our study was comparable with that of the national MIS LLIN use report (40% in the 2015 MIS survey) [14]. However, the overall mean LLIN use (18%) was lower than that of national MIS LLIN use report, which may be due to the difference in study designs. The MIS survey was a cross-sectional study, and did not provide LLIN use information over time. The mean LLIN use over the study period was also much lower than findings from studies in several sub-Saharan African countries [37, 38, 40].

The observed low LLIN use in our study could be related to a reported high attrition rate of the LLINs in the study area [28]. Our previous quantitative study on durability of the same LLINs as this study showed that 61% of LLINs were lost after one year and 96% after two years of follow-up [28]. Two main factors were mentioned as possible causes for this high attrition rate of LLINs. The first was unexpectedly dry and warmer climatic conditions following the El Nino effect in 2015 [27], manifested by a decline in annual rainfall (by 60% in 2015) and an increase in average maximum temperature (2°C above normal) [44]. At this period a parallel study showed low mosquito abundance and low human biting rates [45]. In addition to this, the positivity rate for *Plasmodium* species was zero for mosquito specimen tested for sprozoites [45]. A decline in malaria incidence (only 37% of pre-distribution incidence) was also documented in the study area [26]. This may lead LLIN users to a lower perceived risk of nuisance from mosquitoes and malaria infection. The second reason was the LLIN users’ behaviour and perception toward the serviceable life cycle of the LLINs [33]. A qualitative study on the same LLINs of this study showed that many informants believe that the LLINs would not serve more than one year, by claiming the LLINs lose their insecticide effect after six months (by mentioning the “nets stopped killing bugs”) [33]. Moreover, they mentioned that after one year most of the LLINs were used for other purposes, such as grain storage and transportation from the field, toilet covers, blankets, bed sheets and mattress covers [33].

In this study, the age-specific difference in LLIN use was observed and remained unchanged over the study period. The older age groups over 25 years were using LLINs more often than those of more vulnerable age groups, such as children less than five years, as has been observed elsewhere in Ethiopia [15, 46]. The reasons for a lower LLIN use by more vulnerable age groups need to be further investigated. Unlike our findings, a Ugandan study reported a higher LLIN use among vulnerable age groups [47]. This may be due to a higher malaria prevalence and incidence rate among children in Uganda [48, 49] compared with our study area [34].

The wealth status of households was not significantly associated with LLIN use. This finding is similar to the study findings from southern Ethiopia, northern Nigeria and Uganda [15, 50, 51]. Some studies show a significant association between wealth status and LLIN use in which people living in wealthy households were more likely to use LLINs compared to the poor [52, 53]. In contrast to this, some other studies have shown that people living in poor households were more likely to use the LLINs compared to the wealthy [54, 55]. The lack of significant associations between the wealth status and LLIN use in our study may be influenced by context of the study as a decline in mosquito populations and malaria incidence following unexpected dry and warm weather condition in the study period may commonly affect all households irrespective of their wealth status.

A study from southern Ethiopia reported that LLIN use decreased with an increasing distance from the vector breeding site [15]. However, we did not found a significant difference between residents who lived within and more than 1 km away from Lake Zeway or the Bulbula River, which have been identified as the locations where most breeding sites are found [22, 23]. Having a large family size was associated with a lower use of LLINs, with a similar finding observed in southwest Ethiopia [56]. The reason for this could be an inadequate number of LLINs for households with a large family size due to a high attrition of LLINs in our study area [28].

Achieving malaria elimination in Ethiopia in 2020 requires maintaining high coverage and consistent use of LLINs throughout all season of the year. However, our study findings suggest that mere universal LLIN coverage immediately post-distribution of LLINs does not guarantee for required level of LLIN use. Therefore, the national public health policymakers may use these findings to revise ongoing LLIN distribution schedules and communication and advocacy activity regarding LLIN ownership and use. The behavioural aspects that determined the ownership and use of LLINs should be taken into consideration during communication and advocacy activity. Behaviour change communication (BCC) messages should be provided on how to handle, hang and use LLINs for only intended purpose. Moreover, children under five years need special attention. The national malaria control programme should encourage the consistent use of the LLIN among under five children by involving children’s parents and caregivers even in areas or seasons of low mosquitos and malaria transmission.

Our study had a number of limitations: 1) The LLINs use data were collected based on self-reporting. This may have led to social desirability bias as people prefer a “yes” response. To minimize the bias, the respondents were asked to list the name of the household members who used a LLIN the night before the date of interview. Furthermore, the data collectors observed whether the LLINs were hung over the bed or not. 2) There may be possible interviewer fatigue and reporting bias due to frequent weekly visits for relatively longer period of time by anticipating the next week visit. This may increase the actual LLIN use than would be expected without intensive follow-up or it would be still possible social desirability bias as people prefer to report use instead of reporting non-use. However, because the LLIN use was much lower than expected in the study, this potential limitation is less likely to have influenced the results. 3) There may also be a possible recall bias on the causes of LLIN loss since the data on the reasons for LLIN loss was collected after two years of LLIN distribution. 4) LLIN use was not evaluated for pregnant women, and reasons for not using the available LLINs were not investigated.

## Conclusion

In conclusion, the data showed that despite universal LLIN coverage, a low LLIN ownership and use was observed during 121 weeks of follow-up in the study area. A decline in mosquito populations and malaria incidence following unexpected dry and warm weather condition in the study period could indirectly affect the ownership and LLIN use by decreasing the perceived risk of mosquito bites and malaria infection. However, a high attrition rate of LLINs is the primary reason for an observed low LLIN ownership and use. Meanwhile, age groups, educational status of the head of the household and family size were the main predictors of LLIN use. Consequently, addressing the causes of early loss of LLINs from the household would help to maximize LLIN ownership and use. Since more than 90% of LLINs were lost within two years after LLIN distribution, LLIN replacement strategies should be strengthened to ensure maximal health benefits to the community. Last, the reasons for lower LLIN use by more vulnerable age groups need to be investigated.

## Supporting information

S1 File Questionnaire used to conduct census on selected socio-demographic variables and to gather data on malaria prevention and treatment practices in Adami Tullu District in south-central Ethiopia. (PDF)

S2 File Questionnaire used to conduct weekly LLIN use data collection in Adami Tullu District in south-central Ethiopia, October 2014 –January 2017. (PDF)

S3 File Raw data used to construct Figs 3, 4, 5 and 6 in Adami Tullu District in south-central Ethiopia, October 2014 –January 2017. (XLSX)
