# Supplementary material for: Low use of long-lasting insecticidal nets for malaria prevention in south-central Ethiopia: A community-based cohort study
Source: PLoS One. 2019 Jan 10;14(1):e0210578. doi: 10.1371/journal.pone.0210578 (PMC6328101; doi:10.1371/journal.pone.0210578)
Supplement: S1 File — (PDF) [file pone.0210578.s001.pdf]

QUESTIONNAIRE TO CONDUCT CENSUS ON SELECTED SOCIO-DEMOGRAPHIC VARIABLES AND TO GATHER DATA ON MALARIA PREVENTION AND TREATMENT PRACTICES

General Information

**GI1**	Household number	____________________
**GI2**	Site in which the interview is being conducted	a) Kebele _______________________b)Zone_______________________<br>c)Gare ___________________________
**GI3**	Personnel (name and signature)	a) Interviewer____<br>b) Supervisor___________________________________
**GI4**	Date of visit	[_____|_____| ______| dd | mm| yyyy

Introduction and Consent

My name is___________ and I'm working for Hawassa University and Addis Ababa University. We are conducting a survey about malaria in collaboration with the Woreda Health Office. We would very much appreciate your participation in this survey. This information will help the Oromia Regional Health Bureau to plan health services. This interview could take less than 15 minutes to complete. Whatever information you provide will be kept strictly confidential and will not be shown to other persons. Participation in this survey is voluntary and you can choose not to answer any individual questions or all of the questions. However, we hope that you will participate fully in this survey since your views are important. There will be weekly visit for the next 2 years.

Do you have any questions about the survey? May I begin the interview now?

Verbal consent given to interview, check box ☐

Section 1: Household members' listing and socio-demographic and economic characteristics											
**Q101**	Total number of household members	Number ___________									
**Start listing from the respondent him/herself**											
**Q102a**		**102b**	**102 c**	**102d**	**102e**	**102f**	**102g**	**102h**	**102i**	**102j**	**102k**
**Individual ID**	**Household Members**	**Age**	**Sex**	**Relationship to head of household**	**Educational status**	**Marital Status**	**Current pregnancy status 1. Yes 2. No**	**Duration of pregnancy in months**	**Occupation**	**Ethnicity**	**Religion**
1											
2											
3											
4											
5											
6											
7											
8											
9											
10											
11											
12											
13											
14											
15											

Sex
1. Male
2. Female

Relationship
1. head
2.Wife or husband
3. Child;
4. Relative
5. Maid;
6. Other

Educational Status
(6 years and above)
- **I=** Illiterate
- **RW=** Read and Write only
- If formal education, write the highest grade Completed

Marital Status(15years & above)
1.Maried
2.Living together
3.Divorced or separated
4.Widowed
5. Naver married/naver lived together

Occupation
(18 years and above)
1. Employed
2. House wife
3. Farmer
4. Day laborer
5. Trader
6. Fishery
7. Student
8. No job/dependent
9. Housemaid
10. Others

Ethnicity
1.Oromo
2. Amhara
3. Gurage
4. Other (Specify__)

Religion
1.Orthodox
2.Muslim
3.Protestant
4.Other (Specify__)

**Q103**	Does your household have:<br>Electricity?<br>A watch?<br>A radio?<br>A television?<br>A mobile telephone?<br>A non-mobile telephone?<br>A refrigerator?<br>A table?<br>A chair?<br>A bed?<br>An electric mitad?<br>A kerosene lamp/pressure lamp?	Yes No<br>Electricity……………………………1 2<br>Watch……………………………1 2<br>Radio……………………………1 2<br>Television……………………………1 2<br>Mobile Telephone……………………1 2<br>Non-Mobile Telephone………………1 2<br>Refrigerator…………………………1 2<br>Table……………………………1 2<br>Chair……………………………1 2<br>Bed……………………………1 2<br>Electric Mitad……………………1 2<br>Kerosene/Pressure Lamp……………1 2	
**Q104**	Do you have a separate room which is used as a kitchen?	Yes……………………………………1<br>No……………………………………2	
**Q105**	Main material of the floor.<br><br>***(Record observation)***	Earth/Dung ……………………………1<br>Ceramic Tiles…………………………2<br>Cement………. . . . . …………..3<br>Other…………………………………96<br>Specify________________________	
**Q106**	Main material of the roof<br><br>***(Record observation)***	Thatch/Leaf……………………………1<br>Corrugated Iron . . . . ……... ………..2<br>Cement/Concrete . . . . . ……………... . . . . 3<br>Other…………………………………..96<br>(Specify)_____________________	
**Q107**	Main material of the exterior wall.<br><br>***(Record observation)***	No wall…………………………………..1<br>Wood……………………………………2<br>Wood with mud…………………………3<br>Wood with mud and cement………………..4<br>Cement blocks……………………………5<br>Other…………………………………..96<br>(Specify)_____________________	
**Q108**	How many rooms in this household are used for sleeping?	Number of rooms[__|__]	
**Q109**	How many sleeping spaces such as mats, rugs, mattresses or beds are used in this household?		
**Q110**	Does any member of this household own:<br>A bicycle?<br>A motorcycle?<br>An animal-drawn cart?<br>A car or truck?	Yes No<br>Bicycle……………………………1 2<br>Motorcycle……………………………1 2<br>Animal-drawn cart…………………1 2<br>Car/truck……………………………1 2	
**Q111**	Does any member of this household own any land that can be used for agriculture?	Yes……………………………………1<br>No……………………………………2	→ **Skip to Q113**
**Q112**	How many (LOCAL UNITS) of agricultural land do members of this household own?<br>***(If unknown enter 98)***	Local units [___|___]<br>Specify the local unit__________________	
**Q113**	Does this household own any livestock, herds, or farm animals?	Yes……………………………………1<br>No……………………………………2	
**Q114**	How many of the following animals does this household own?<br>Milk cows, oxen, or bulls?<br>Horses, donkeys, or mules?<br>Goats?<br>Sheep?<br>Chickens?<br>***(If unknown, enter 98)***	Milk cows, oxen, or bulls------ [| |]<br>Horses, donkeys, or mules----- [| |]<br>Goats---------------------------- [| |]<br>Sheep---------------------------- [| |]<br>Chickens------------------------ [| |]	
**Q115**	Does any member of this household have an account with a bank/credit association/micro finance?	Yes……………………………………1<br>No……………………………………2	

**Q116**	What is the main source of drinking water for members of your household?<br><br>***(Do not read out Responses)***	**Piped (Tap)**<br>Piped into dwelling……..…1<br>Piped into compound……...2<br>Piped outside compound….3<br>Covered Well…………..…4<br>Protected Spring………..…..5<br><br>**Open Well/Spring**<br>Open Well…………………6<br>Open Spring……………..…7<br><br>**Surface Water**<br>River………………………..8<br>Pond/Lake/Dam…………..9<br>Rainwater……………….10<br><br>Other…..………..………..11<br>Specify____________________	
**Q117**	What kind of toilet facility do most members of your household use?<br><br>***(observe latrine)***	Flush toilet……………….…...………..1<br>Pit latrine/traditional pit toilet..……….2<br>Ventilated improved pit latrine (VIP) …3<br>No facility/Bush/Field… ………….….4<br>Other……….…………………..…..….5<br>Other(Specify)____________________	→ **Skip to Q201** (for 4)
**Q118**	Do you share this facility with other households?	Yes…………….1<br>No..…………...2	

Section 2: Malaria prevention and treatment

**Q201**	Does your household have any mosquito net that can be used while sleeping?	Yes…………….1<br>No..…………..2 →			**Skip to Q211**
**Q202**	How many mosquito nets do your household have?	Number of Nets _________			
**Q203**	Ask respondent to show you the net(s) in the household.	NET #1 ______<br><br>Observed .... 1<br>Not observed......... 2	NET #2 _______<br><br>Observed...............1<br>Not observed 2	NET #3 _______<br><br>Observed.................1<br>Not observed.. 2	
**Q204**	How long ago did your household obtain the mosquito net?	____ ____ Months ago	____ ___<br>Months ago	____ ____<br>Months ago	
**Q205**	Where did you obtain the net?	Government Clinic/hospital Health extension worker……………….1<br>Retail shop Pharmacy…………….2<br>Workplace……………3<br>Other (specify)__________.....4<br>Don't know……………..98	Government Clinic/hospital Health extension worker…………….1<br>Retail shop Pharmacy…………2<br>Workplace…………3<br>Other (specify)_________....4<br>Don't know………98	Government Clinic/hospital Health extension worker…………….1<br>Retail shop Pharmacy…………2<br>Workplace………..3<br>Other (specify.........4<br>Don't know…….98	
**Q206**	Did you purchase the net?	YES............1<br>NO..............2<br>Not sure............ 8	YES............1<br>NO..........2<br>Not sure............ 8	YES............1<br>NO.............2<br>Not sure............ 8	→ skip to 208 (for 2, 8)

**Q207**	How much did you pay for the net when it was purchased?	_________ birr	_________ birr	_________ birr	
**Q208**	Did anyone sleep under the mosquito net last night?	Yes……………1<br>No..…………...2<br>Not sure………8	Yes……………1<br>No..…………...2<br>Not sure………8	Yes……………1<br>No..…………...2<br>Not sure………8	**Skip to Q210**
**Q209**	Who slept under this mosquito net last night?	Individual ID<br>1. __________<br>2. __________<br>3. _________<br>4. ________	Individual ID<br>1. __________<br>2. __________<br>3. _________<br>4. _________	Individual ID<br>1__________<br>2__________<br>3_________<br>4_________	
**Q210**	Why did no-one sleep under this mosquito net last night?	No malaria………….. 1<br>No nuisance/insects… 2<br>No space for net ……….3<br>Irritation ………………4<br>Suffocation / too hot ….5<br>Difficult hanging net ….6<br>Shape ………………..7<br>Absence from home …..8<br>Other.......................... 9<br>Don't know……....98	No malaria………. 1<br>No nuisance/insects.. 2<br>No space for net ….3<br>Irritation …………4<br>Suffocation / too hot ..5<br>Difficult hanging net ..6<br>Shape …………..7<br>Absence from home ..8<br>Other......................... 9<br>Don't know……….98	No malaria….. 1<br>No nuisance/insects.. 2<br>No space for net …3<br>Irritation …………4<br>Suffocation / too hot ...5<br>Difficult hanging net ..6<br>Shape ………………..7<br>Absence from home…..8<br>Other.......................... 9<br>Don't know……….98	
**Q211**	Has your house ever been sprayed with insecticide for malaria prevention by spraymen from the District Health Office?	Yes……………1<br>No...…………...2<br>Not sure..………8			**Skip to Q215**
**Q212**	How many months ago was your house sprayed?<br>***(If less than one month, record 0)***	Months ago [___/___]<br>Not sure…..8			
**Q213**	At any time in the past 12 months, have the walls in your dwelling been plastered or painted?	Yes... ...1<br>No... 2			
**Q214**	How many months ago were the walls plastered or painted? *If less than one month, record 0.*	MONTHS AGO , _____ _____			
**Q215**	Was there death of family member in the last one year?	Yes……….1<br>No...……...2	→ When did it occur?<br>________months ago	Sex<br>Male……1<br>Female…2	Age<br>______<br>**Year/Month**

Health service seeking and utilization									
**ID**	**Q216**	**Q217**	**Q218**	**Q219**	**Q220**	**Q221**	**Q222**	**Q223**	**Q224**
	Have any of your family members faced any health problem during the **last 2** months?<br>**Yes……1**<br>**No…….2 ► Q222**	What was the sickness/ injury faced?<br>Malaria……….1<br>Diarrhea……..2<br>Injury………..3<br>Dental……….4<br>Opthalmic……5<br>Skin disease……6<br>Ear/nose/throt (ENT)….7<br>Tuberclosis…….8<br>Other (specify)________	For how many days were he/she absent from usual activity due to the health problem during the **last 2** months?	Has he/she received medical assistance or consulted from health institutions or traditional healers during the **last 2** months?<br>**Yes……1**<br>**No…….2 ► Q221**	Where did he/she receive or consult medical assistance primarily?<br>Hospital…………………1<br>Health center………….2<br>Health post……………..3<br>Private Clinics………….4<br>Private Pharmacy……..5<br>Traditional healer……..6<br>Religious/spiritual……7<br>Other (specify)…………8	What was the main reason for he/she not to consult health institutions/ traditional healer during the **last 2** months?<br>Lack of money…..1<br>Expensive………2<br>Too far ……….3<br>Do not believe in medicine……….4<br>Lack of health professional……5<br>Poor quality/ service……….6<br>Did not require medical assistance. 7<br>Other (specify)………8	Have any of your family member consulted any medical assistance during the **last 12** months? (Regardless of whether sick or not)?<br>**Yes……1**<br>**No…….2**	How many times have he/she consulted any medical assistance during the **last 12** months?	Has member of your family been ill with a fever at any time in the last 7days?<br>**Yes……1**<br>**No…….2**
1									
2									
3									
4									
5									
6									
7									
8									
9									
10									
11									
12									
13									
14									
15									

GAAFFIIWWAN QORANNOO LAKKOOBSA UUMMATAA HAWAASAA FILATAMANII FI RAGAALEE ITTISAA FI YAALII DHUKKUBA BUSAA QORACHUUF QOPHAA'E

Odeeffannoo Walii Galaa		
**GI1**	Lakk. (Koodii) Mana	____________________
**GI2**	Dirree itti gaaffii fi deebiin geggeeffame	a) Ganda _________________________ b) Zone_______________<br>c)Garee ___________________________
**GI3**	Personeelii (Maqaa fi Mallattoo)	a) Gaaffii kan gaafate ______________________________<br>b) To'ataa Dirree Supervis__________________________________
**GI4**	Guyyaa Hordoffii	[_____|_____| ______| Guyyaa | Ji'a | Bara

Seensaa fi Walii Galtee

Maqaan koo __________________ jedhama. Hojjaa kanaaYuunivarsiitii Addis Ababaa fi Hawwaasaa finii hojjedha. Waajjira fayyaa aanaa keessanii wajjin wal ta'uudhaan qorannoo dhukkuba busaa irratti hojjechaa jira. Qorannoo kana irratti hirmaannaa gootaniif dursinee isin galateeffanna. Bu'aan qorannoo kanaa Biiroon Eegumsa Fayyaa Oromia karoorsanii tajaajila adda addaa kennuuf gargaara.

Gaaffii fi deebiin kun daqiiqaa15 keessatti xumurama. Odeeffannoon isin nuu kennitan kamiyyuu icciitii cimaadhaan kan eegamuu fi qaama kamitti iyyuu kan hin agarsiifane ta'a. Hirmmaannaan isin gaaffii if deebii kana irratti gootan fedha keessan irratti kan hundaa'e yammuu ta'u gaaffii kamiyyuu filattanii deebisuu yookin gaaffii hunda iyyuu deebisuu dhiisuudhaaf mirga guutuu qabdu. Haa ta'u iyyuu malee deebiin isin nuu keennitan qorannoo kanaaf baayyee murtteessaa waan ta'eef gutumaan guutuutti gaaffii fi deebii kana irratti hirmaattu jennee abdii guddaa qabna.

Akkasumas Waggaa lamaan dhufanniif torban torbaniin du'annaan kan deemsifamu ta'a. Qorannaa kana ilaalchisee gaaffii qabduu?Gaaffii fi deebii keenya itti fufuu dandeenyaa?

Gaafi gaafatamu deebi kennudhaf eyyamamadha yoo ta'e, sanduqa kessatti mallato godhii ☐

Kutaa1: - Ibsa Haala Hawaas-Dinagdee , Halaa Uummataa fi Miseensota Maatii										
**Q101**	**Baayyina Miseensota Maatii**	**Baayyina __________**								
**Nama Gaafatamaa jiru irraa eegalaatii tarreessaa**										
**Q102a**		**102b**	**102c**	**102d**	**102e**	**102f**	**102g**	**102h**	**102i**	**102j**	**102k**
Kodii dhunfaa	**Miseensota Maatichaa**	**Umurii**	**Saala**	**Walitti dhufeenya**	**Sadarkaa barnootaa**	**Sadarka matii 1.Eyee 2. Lakki**	**Ulfa yeroo amma/kan ulfa tee jirra**	**Ji'a meqaaf ulfofte**	**Hojii**	**Lammii/ Qomoo**	**Amantii**
1											
2											
3											
4											
5											
6											
7											
8											
9											
10											
11											
12											
13											
14											
15											

Saala
1. Dhiira
2. Dhalaa

Walitti dhufeenya
1. Abbaa waara /hadha waara
2. Haadha waara
3. Mucaa
4. Fira
5. Hojjettuu manaa
6. Kan biroo

Sadarkaa barnootaa (waggaa 6 fi ol)
- **I=** Hin baranne
- **RW=** Barreessuu fi dubbisuu
- Barnoota idilee yoo ta'e sadarkaa isaa barreessi

Sadarka matii(waggaa 15 ol)
1.ka fudhe
2.wajjin kanjiratan
3. Walihikan/gare gare kanbaan
4. ka iradue
5. ka hinfune;

Hojii (waggaa 18 fi ol)
1. Hojj. Mootummaa
2. Haadha manaa
3. Qotee bulaa
4. Hojii oolmaa
5. Daldalaa
6. Qurxummi**i** qabduu
7. Barataa
8. Hojii hin qabuu
9. Hojjettuu manaa
10. Kan biroo

Lammii/ Qomoo
1.Oromoo
2. Amaara
3. Guraagee
4. Kan biroo (Ibsi__)

Amantii
1. Ortodoksii
2. Islaama
3. Pirotestantii
4. Kan biroo (Ibsi__)

Q103	Mana keessanitti tajaajila armaan gadii qabduu?<br>Elektirikaa?<br>Sa'atii?<br>Televizhinii?<br>Raadiyoo ?<br>Mobaayilii?<br>Bilbila manaa?<br>Firiijii?<br>Teessuma?<br>Miinjaala<br>Siree ciisichaa?<br>Eelee elektirikaa?<br>Buttaa gaazii?	Eyyee Lakkii<br>Elektirikaa ………….………….…….1 2<br>Sa'atii ……………………………….……1 2<br>Televizhinii……………..…...…….1 2<br>Raadiyoo ………………………..…….1 2<br>Mobaayilii ……..………….…..1 2<br>Bilbila manaa …………..…..1 2<br>Firiijii.. …..……….1 2<br>Miinjaala..……….….…….1 2<br>Teessuma ……………………….……...1 2<br>Siree ciisichaa ………………..………1 2<br>Eelee elektirikaa ………...……..….1 2<br>Buttaa gaazii ………..…...1 2	
Q104	Mana nyaata itti bilcheeffattan kophaatti qabduu	Eyyee ……………………………………………1<br>Lakkii …………………………………………….2	
Q105	Lafti mana keessanii maal irraa hojjeteme?<br>(Waan daawwatte sana waraabi)	Biyyoo.…….….……………………...1<br>Seraamikii… …….………………………..2<br>Simmintoo …... . . . . ………..…..3<br>Kan biroo ………………………………96<br>(Kan biroo Ibsaa)_______________________	
Q106	Guutuun (Xaaraan) mana keessanii maal irraa hojjeteme?<br>(Waan daawwatte sana waraabi)	Baala/ fi Citaa…………………………………1<br>Qorqorroo ……… . . . . ……..... ……..…..2<br>Simmintoo….…. . . . . ……………... . . . . 3<br>Kan biroo ……………….………………..96<br>(Kan biroo Ibsaa)_____________________	
Q107	Dhaabin mana keessanii (Girgiddaa) maal irraa hojjeteme?<br><br>(Waan daawwatte sana waraabi)	Hin qabu ……………………………………..1<br>Muka ……………………………………….2<br>Mukaa fi dhoqqee …………………………….3<br>Muka ,dhoqqee fi simmintoo ………………..4<br>Bilookeetii simmintoo ……………………...5<br>Bilookeetii Biyyoo…………………………..6<br>Kan biroo ……………….……………..96<br>(Kan biroo Ibsaa)________________	
Q108	Mana keessan keessaa kutaa ciisicha meeqa qabdu?	Lakkoobsaa kutaa[_______|______]	
Q109	Maatiin kun edoo ciisicha kan akka firashii, siree, minxaafii yookiin boraatii meeqatti fayyadama?	Lakkoobsaa edoo ciisicha [_____|________]	
Q110	Maatii mana kanaa keessaa namni armaan gadii kana qabu jiraa:<br>Saayikilii?<br>Dhokdhokkee?<br>Gaarii?<br>Konkolaataa?	Eyyee Lakkii<br>Saayikilii …….….………...1 2<br>Dokdhoqqee ……….….……1 2<br>Gaarii …..………………..1 2<br>Konkolaataa …………...…..1 2	
Q111	Maatii mana kanaa keessaa namni lafa qonnaa qabu jiraa?	Eyyee ………………………………………1<br>Lakkii ……………………………………….2 →	**Q113tti ce'i**
Q112	Maatiin kun lafa qonnaa safartuu hammame qabu?<br>*(*Yoo hinbeekamu ta'e "98" barreessi)	Safartuu lafaa naannoo kanaatti fayyadamuudhaan ibsaa ________________<br><br>Safartuu lafaa gargaramitan ibsa _________	
Q113	Maatiin kun horii manaa qabuu?	Eyyee …………………………………………1<br>Lakkii ………………………………………….2	

**Q114**	Maatiin kun horii manaa armaan gadii meeqa qabu?<br>Loon annanii, Qotiyyoo, Horii foonii?<br>Farda, Harree, Gaangee?<br>Re'ee?<br>Hoolota?<br>Lukkuuwwan?<br>(Yoo hinbeekamu ta'e "98" barreessi)	Loon annanii, Qotiyyoo, Horii foonii------- | | | Farda, Harree, Gaangee --------- | | | Re'ee ------------------- | | | Hoolota ----------------- | | | Lukkuuwwan -------------- | | |	
**Q115**	Maatii kana keessaa namni herrega qusannaa baankii qabu jiraa?	Eyyee ……………………………………………1<br>Lakkii …………………………………………….2	
**Q116**	Maddi bishaan dhugaatii maatii keessanii maali?<br>(Deebicha hin dubbisin)	*Boombaa*<br>Boombaa mana keessatti ….….1<br>Boombaa mooraa keessatti ……...2<br>Boombaa mooraa alatti ……..….3<br>Biirii eegumsa qabu …………..…4<br>Burqituu eegumsa qabu ……….…..5<br><br>*Biirii banamaa/burqituu*<br>Biirii banaa …………………6<br>Burqituu banaa ………………7<br>*Bishaan Yaa'u*<br>Bishaan lagaa ……………..8<br>Kuufama/haroo ……………9<br>Bishaan bokkaa ………….10<br>Kan biroo ……………………..11<br>(Kan biroo Ibsaa)______________________	
**Q117**	Maatiin keessan mana fincaanii attamii gargaaramu?<br><br>(Mana fincaanichaa daawwaadhu)	Boolla fincaanii bishaannin kan hojetu (Flush toilet)……………..1<br>Boolla fincaanii kan duri /traditional pit toilet..……2<br>Mana fincaanii (Ventilated improved pit latrine) -3<br>Hin qabu / Bosona keessatti /Bakkeetti..........4 →<br>Kan biraa …………………………………5<br>Kan biraa (ibsaa)______________________	**Q201tti ce'i**
**Q118**	**Mana fincaanii kana maatii biraa waliin gargaaramtuu?**	**Eyyee …………….1**<br>**Lakkii ..…………...2**	

<u>Kutaa: 2ffaa</u> Ittisaa fi Yaalii Dhukkuba Busaa

Q201	Mana kana keessaa tajajila kenu kandandu agoobara qabduu?	Eyyee ……………1<br>Lakkii ..…………...2 →			**Q211 itti ce'i**
Q202	Agoobara meeqa qabduu?	Lakkobsaa Agoobaraa_____________			
Q203	Agoobara mana keessa jiru akka agarsiisaniif gaafatamtoota gaafadhu.	Agoobara #1	Agoobara #2	Agoobara #3	
		Ilaalameera .... 1<br>Hin ilaalamne...... 2	Ilaalameera ....1<br>Hin ilaalamne 2	Ilaalameera ....1<br>Hin ilaalamne 2	
Q204	Maatiin kun agoobara bookee busaa ittisu yoom argate?	_____ _____Ji'a	____ ___ Ji'a	____ ____ Ji'a	
Q205	Agoobaricha eessaa argattan?	Mootummaa<br>Kilniika/ Hospitaala<br>Hojjettuu Eksteenshenii Fayyaa......................1<br>Faarmaasii dhuunfaa......2<br>Bakka hojii……......…3<br>Kan biroo (Ibsi)............4<br>Hinbeeku…………....98	Mootummaa<br>Kilniika/ Hospitaala<br>Hojjettuu Eksteenshenii Fayyaa…................1<br>Faarmaasii dhuunfaa....2<br>Bakka hojii………3<br>Kan biroo (Ibsi)...........4<br>Hinbeeku……….98	Mootummaa<br>Kilniika/ Hospitaala<br>Hojjettuu Eksteenshenii Fayyaa…................1<br>Faarmaasii dhuunfaa...2<br>Bakka hojii………3<br>Kan biroo (Ibsi)...........4<br>Hinbeeku……….98	

Q206	Agoobaricha ni bittanii?	Eyyee............1<br>Lakkii..............2<br>Sirritti hin beeku...8	Eyyee............1<br>Lakkii..............2<br>Sirritti hin beeku..8	Eyyee............1<br>Lakkii(8)..............2<br>Sirritti hin beeku....... 8	Q208 tti darbi
Q207	Agoobaricha qarshii meeqaan bittan?	Qarshii _________	Qarshii _________	Qarshii _________	
Q208	Galgala darbe/eda namni agoobara hidhatee rafe jiraa?	Eyyee …………….1<br>Lakkii..…………...2<br>Sirritti hin beeku...8	Eyyee ……….1<br>Lakkii..……..2<br>Sirritti hin beeku..8	Eyyee …………….1<br>Lakkii..…………...2<br>Sirritti hin beeku...8	**Q210 ce'e**
Q209	Eenyutu agoobara hidhatee rafe?	Koddi dhunfa<br>1.____________<br>2.____________<br>3.____________<br>4.____________	Koddi dhunfa<br>1.____________<br>2.____________<br>3.____________<br>4.____________	Koddi dhunfa<br>1.______________<br>2.______________<br>3.______________<br>4.______________	
Q210	Kaleessa halkan (eda galgala) maatii kana keessaaa namni kamiyyuu agoobara maaliif hin huffanne?	Busaan waan hinjirreef..................... 1<br>Ilbiisonni/bookeen waan hinjirreef..................... 2<br>Agoobaraaf bakki waan hinjirreef ....3<br>Nama rifachiisa …..........4<br>Namatti o'a/ bulluqa ...5<br>Fannisuuf waan ulfaatuuf ....6<br>Bochi isaa hin mijatu...7<br>Mana keessa agoobarri waan hin jirreef….......8<br>Sababa biroo............... 9<br>Hin beeku……...........98	Busaan waan hinjirreef................ 1<br>Ilbiisonni/bookeen waan hinjirreef....... 2<br>Agoobaraaf bakki waan hinjirreef ....3<br>Nama rifachiisa.....4<br>Namatti o'a/ bulluqa.......................5<br>Fannisuuf waan ulfaatuuf …............6<br>Bochi isaa hin mijatu..7<br>Mana keessa agoobarri waan hin jirreef….......8<br>Sababa biroo............. 9<br>Hin beeku............98	Busaan waan hinjirreef.................... 1<br>Ilbiisonni/bookeen waan hinjirreef.......................2<br>Agoobaraaf bakki waan hinjirreef ....3<br>Nama rifachiisa ....4<br>Namatti o'a/ bulluqa .....5<br>Fannisuuf waan ulfaatuuf …....................6<br>Bochi isaa hin mijatu..7<br>Mana keessa agoobarri waan hin jirreef............8<br>Sababa biroo............ ...9<br>Hin beeku……...........98	
Q211	Hojjettoota fayyaa aanaa keessaniin qorichi farra bookee busaa mana keessanitti ni biifamaa?		Eyyee …………….1<br>Lakkii..…………...2<br>Hin yaadadhu(Hin beeku).……….3		**Q215 tti darbi**
Q212	Ji'a meeqa dura qorichi kun kan isinii biifame?<br>(Ji'a tokko gadi yoo ta'e waraabi 01)		Ji'a [___/___]<br>Hin yaadadhu/…..98		
Q213	Batiiwwan 12 darban kam iyyuu keessatti dhabaani /Girgida/ mana keessanii dibamee yookiin lastikiin itti godhamee beekaa?		Eyyee.. ...1<br>Lakkii.. 2		
Q214	Batiiwwan meeqa dura dhabaani /Girgida mana keessanii kan dibame yookiin lastikiin kan itti godhame? (Ji'a tokko gadi yoo ta'e waraabi 01)		Ji'ota/baatiiwwaniin dura , _____ _____		
Q215	Waggaa kana keessa mana kana keessaa namni boqate/ du'e jiraa?	Eyyee ….1 →<br>Lakkii ..….2	Yoom boqote?<br>Ji'a meeqa<br>dura ____	Saala<br>Dhira …1<br>Dhala ..…2	**Umurii ______ Waggaa / Ji'a**

Fedhii fi itti fayyadama tajaajila fayyaa									
**kodii**	216	**217**	**218**	**219**	**220**	**221**	**222**	**223**	**224**
	**Baatiwwan 12 darban keessatti namni maatii keessan keessaa dhibeen fayyaa isa mudate jiraa**<br>Eyyee……1<br>Lakkii…2 ► **Q222**	Dhibee yookin balaa /madaa akkamiitu mudate?<br>Busaa……….1<br>Garaa kaasaa……..2<br>Balaa /madaa …......3<br>Dhibee ilkaanii.......4<br>Ija…....................5<br>Dhukuba gogaa…....6<br>Gurra/funyaan/liqimsituu(ENT)…..........7<br>Dhukkuba sombaa…............8<br>Kan biroo(ibsi)_____	Baatii lamaan darban keessatti namni dhukabsate sun guyyaa meeqaaf hojii irraa hafe?	Baatii lamaan darban keessatti namni dhukabsate sun dhabbilee fayyaa irraa yookiin aadaadhaa iraa gargaarsa fayyaa argatee jiraa?<br>Eyyee……1<br>Lakkii…2► **Q221**	Gargaarsa fayyaa kana adda dureedhaan eessa irraa argata/ti?<br>Hospitaala irraa.................1<br>Buufata fayyaa irraa...........2<br>Kellaa fayya..................3<br>Kilnika dhuunfaa irraa............................4<br>Faarmaasii dhuunffaa..................5<br>Qoricha aadaa.............6<br>Dhaabbata amantii.....7<br>Kan biroo(ibsi)……...8	Baatii lamaan darban keessatti namni dhukabsate sun dhabbilee fayyaa yookiin aadaa iraa gargaarsa/ gorsa fayyaa akka hin arganneef sababni guddaan maal ture?<br>Maallaqa dhabuu.....1<br>Gatiin yaala qaala'uu /miyaa'uu isaaa....2<br>Baayyee fagoo ....3<br>Qorichatti hin amanu/tu............4<br>Ogummaa fayyaa dhabuu.........5<br>Tajaajila qulqulina hin qabne....6<br>Gargaarsi fayyaa waan hin barbaachifneef. ....7<br>Kan biroo(ibsi)_______..8	Baatii 12 darban keessatti maatii kana keessaa namni gargaarsi faayyaa kennameef jiraa?(Dhukkubsates dhukkubsachuu baatus)<br>Eyyee….1<br>Lakkii….2	Baatii 12 darban keessatti ala meeqa gargaarsa fayyaa argate/te?	Guyyaa torban darban keessatti maatii kana keessa namni dhukuba ho'aa qaaman qabamee jiraa?<br>Eyyee….1<br>Lakkii…2
1									
2									
3									
4									
5									
6									
7									
8									
9									
10									
11									
12									
13									
14									
15									
