# Supplementary material for: Low use of long-lasting insecticidal nets for malaria prevention in south-central Ethiopia: A community-based cohort study
Source: PLoS One. 2019 Jan 10;14(1):e0210578. doi: 10.1371/journal.pone.0210578 (PMC6328101; doi:10.1371/journal.pone.0210578)
Supplement: S2 File — (PDF) [file pone.0210578.s002.pdf]

MalTrials Project: Weekly malaria data collection format				
Kebele____________________<br>Gare______________________<br>House number_____________		Date of visit[_____|_____|______]<br>dd | mm| yyyy		
Data collector		Name ________________________ Signature ________________		
**Q01**	Did anyone sleep under the bed net last night?	Yes…………….1<br>No..……………2<br>Not sure………3<br>The household doesn't own net…..4	No, Not sure, The household doesn't own net → **Skip to Q03**	
**Q02**	Who slept under the bed net last night?<br>(List the names)	**1.**________________<br>**2.**________________<br>**3.**________________<br>**4.**________________<br>**5.** ________________	**6.** ____________________<br>**7.**____________________<br>**8.** ____________________<br>**9.** ____________________<br>**10.** ____________________	
**Q03**	Presence of fever any time in the last 48 hours	Yes………1<br>No……….2	If 2 → End	
**Q04***	If there is a member of the family who is febrile during the interview and did not take any medication, take axillary temperature and record the case's name and house number on your note book, then send the case with a referral slip to nurse as soon as possible.<br>***(Use another format if you got more than three case in the same household and attach) (Use separate referral slip for each case)***	Name________________<br>Age _____Years/Months<br>Sex<br>Male……1<br>Female…2	Name________________<br>Age _____Years/Months<br>Sex<br>Male……1<br>Female…2	Name________________<br>Age _____Years/Months<br>Sex<br>Male……1<br>Female…2

** Please confirm the referred case gave blood sample at the health post and if so, request the Nurse to have her/his signature on your note book at the end of the day. The Nurse is expected to label the slide with **the date** and **the case's first name, age, sex and house number**.*

<table>
<tr><th colspan="5">Projektii ‘MalTrials’: Unka Galmee Ragaa Dhukkuba Busaa Torbanii (Afan Oromo)</th></tr>
<tr><td colspan="3">Ganda ________________
Zone__________________
Garee ________________
Lakkophsa manaa_(koodi)_______</td><td colspan="2">Guyyaa Hordoffii [_____|_____|______]
Guy | ji’a | bara

Maqaa **Nama Gaafatamee** ____________________________</td></tr>
<tr><td colspan="2">Ragaa kan Funaanu</td><td colspan="3">Maqaa _____________________ Mallattoo ________</td></tr>
<tr><td>**Q01**</td><td>Galgala darbe namni agoobara hidhaatee rafe jiraa?</td><td colspan="2">Eyyee……………1
Lakkii..…………...2
Hin beeku………3
Agoobara hin qaban…..4</td><td>(2, 3, 4) → **Q03 darbi**</td></tr>
<tr><td>**Q02**</td><td>Eenyutu agoobara hidhatee rafe

(*maqaa tarreessaa*)</td><td colspan="3">**1.**______________________________ **6.**______________________________
2.______________________________ **7.**______________________________
3.______________________________ **8.**______________________________
4.______________________________ **9.**______________________________
5. ______________________________ **10.**_____________________________</td></tr>
<tr><td>**Q03**</td><td>Sa'ati 48 darbe kessaatii namni dhukkuba ho’aa qamaan qabamee jira?</td><td colspan="2">**1.** Eyyee
2. Lakkii →</td><td>Ta’e dhabii</td></tr>
<tr><td>**Q04**[*]</td><td>Yeroo gaaffii fi deebii geggeessitan yoo namni dhukuba ho'aa qaaman qabamee fi Qoricha kan hin gargaaraminii isin quunname galmeessaatii gabaasaa. (Yoo bayyatan 3ol ta’an unka biraaa gargaaramaa)</td><td>Maqaa:______________
____
Umr ____ wagaa/ji’a
Salaa
Dhira.........1
Dhala......…2</td><td>Maqaa:______________
____
Umr ____ wagaa/ji’a
Salaa
Dhira.........1
Dhala......…2</td><td>Maqaa:______________
____
Umr ____ wagaa/ji’a
Salaa
Dhira.........1
Dhala......…2</td></tr>
</table>

[*] *Nami kara keellaa fayyaatti dhiiga kennuf ergame, dhiiga kennu isaa mirkaneefadhu. Yoo dhiigni kennameera ta’e, Nurseichi akka kitaaba yadanno kee irratti simalateesu goodhi Nursein slidi irratti gayyaa,maqaa, umuri, salaa fi lakkoofsa mana haa barressu.*
